# Advanced Imaging of Multiple Myeloma Bone Disease

**DOI:** 10.3389/fendo.2018.00436

**Published:** 2018-08-07

**Authors:** Barry G. Hansford, Rebecca Silbermann

**Affiliations:** ^1^Department of Diagnostic Radiology, Oregon Health and Sciences University, Portland, OR, United States; ^2^Division of Hematology and Medical Oncology, Oregon Health and Sciences University, Knight Cancer Institute, Portland, OR, United States

**Keywords:** myeloma bone disease, MRI imaging, PET imaging, CT imaging, lytic bone disease

## Abstract

Multiple myeloma (MM), a malignancy of mature plasma cells, is the second most common hematologic malignancy and the most frequent cancer to involve the skeleton (1, 2). Bone disease in MM patients is characterized by lytic bone lesions that can result in pathologic fractures and severe pain. While recent advances in MM therapy have significantly increased the median survival of newly diagnosed patients (3), skeletal lesions and their sequelae continue to be a major source of patient morbidity and mortality and bone pain is the most frequent presenting symptom of MM patients (4). Rapid improvements in imaging technology now allow physicians to identify ever smaller skeletal and bone marrow abnormalities, however the clinical value of subtle radiographic findings is not always clear. This review summarizes currently available technologies for assessing MM bone disease and provides guidance for how to choose between imaging modalities.

## Introduction

Multiple myeloma (MM) bone disease continues to be one of the most devastating complications of MM and is characterized by an uncoupling of the normal bone remodeling process. In contrast to physiologic bone remodeling, where bone formation occurs at sites of bone resorption, MM bone disease (MMBD) is marked by local areas of increased osteolysis in areas adjacent to MM cells and highly suppressed or absent osteoblast function. This combination leads to lytic bone lesions that do not heal and generalized bone loss. Pain related to bone destruction occurs in more than two-thirds of patients and is the most frequent symptom at disease presentation. In addition, MMBD results in enhanced tumor growth and fractures, all of which impact survival. Approximately 70% of MM patients have skeletal disease at diagnosis and up to 85% develop bone lesions after diagnosis. Importantly, it is estimated that 60% of patients develop pathologic fractures over their disease course (5, 6), and MM patients with pathologic fractures have a 20% increased risk of death compared to other patients (7). Despite this high prevalence, not all MM patients develop MMBD. For those that do, management of MM bone disease remains a crucial part of their long-term care as MM bone lesions persist in the absence of active disease in the majority of patients.

Skeletal imaging is a critical component of both the initial diagnostic evaluation and the long-term management of patients with plasma cell disorders. MM is preceded by the well-defined conditions monoclonal gammopathy of undetermined significance (MGUS) and smoldering MM (SMM), both of which are currently managed with surveillance. Active (symptomatic) MM is treated with antineoplastic therapy and has historically been diagnosed based on the presence of >10% clonal bone marrow plasma cells or biopsy-proven plasmacytoma in combination with one or more myeloma-defining events: hypercalcemia, renal dysfunction, anemia, and lytic bone disease (referred to as CRAB criteria). The definition of active MM was broadened by the International Myeloma Working Group in 2014 to include patients with a high disease burden based on percentage of monoclonal plasma cell infiltration in the bone marrow or a high serum involved to uninvolved free light chain ratio (>100), as patients with these laboratory biomarkers have a high risk of progression to active disease (8). Importantly, the definition of MM bone disease was clarified to exclude osteoporosis or vertebral compression fractures identified in the absence of other lytic lesions (8). Updated MM diagnostic criteria also included allowance of advanced imaging technology including CT, PET, and MRI for the identification of focal bone lesions, however the decision of which imaging modality to use for which patient remains at the discretion of the provider.

MGUS is an asymptomatic condition defined by the presence of a low concentration (<3 g/dL) serum monoclonal protein, a bone marrow with <10% monoclonal plasma cells, and the absence of CRAB criteria. SMM is defined by the presence of a serum monoclonal protein >3 g/dL and/or 10–60% monoclonal bone marrow plasma cells in addition to the absence of CRAB criteria and an involved to uninvolved serum free light chain ratio <100. MGUS and SMM are premalignant conditions with variable courses. Patients with MGUS and SMM have an overall risk of progression to MM of 1 and 10% per year, respectively (9), however individuals within each group have variable risks of disease progression. Validated multivariate risk models allow prognostic stratification of MGUS and SMM patients into risk categories with 5 year probabilities of MM progression of 2, 10, and 46% for MGUS and 4, 46, and 72% for SMM (10). Interestingly, MGUS and SMM are both associated with osteopenia, altered bone microstructure, and an increased fracture risk (11, 12).

## Pathophysiology of myeloma bone disease

The tightly regulated osteoclast—osteoblast activity of normal physiologic bone remodeling is uncoupled in MM bone disease. MM cells physically disrupt the bone-remodeling compartment, allowing cell-cell contact between MM cells and bone cells and the exchange of soluble factors that mediate the enhanced bone destruction and absent bone formation characteristic of MM bone disease (13). Cellular components of the bone marrow microenvironment, such as osteoclasts, osteocytes, immune cells, and bone marrow stromal cells stimulate the growth and chemoresistance of MM cells in the marrow space through the production of both membrane-bound and soluble growth factors that enhance MM cell growth and increase marrow angiogenesis (14, 15). MM-cell derived cytokines in turn increase osteoclast formation and bone resorption both systemically and in areas of tumor infiltration (16), creating a “vicious cycle” of increased bone resorption leading to increased tumor burden. The bone resorption process itself also results in the release and activation of bone matrix-derived growth factors that further enhance MM cell growth (17). Osteoblast function, in contrast, is highly suppressed or absent, resulting in purely lytic bone lesions.

Skeletal remodeling is abnormal in patients with MGUS and SMM. In retrospective studies, MGUS is associated with a 6 times greater risk of vertebral fracture and 1.4–2.5 times greater risk of any fracture when compared to control populations (11, 18). Limited prospective evaluations of skeletal abnormalities in MGUS have been completed to date, but those that have confirm the high prevalence of vertebral fractures in MGUS and suggest an association between non-traumatic vertebral fractures and a clonal lambda light chain predominance (19). MGUS and SMM are associated with osteopenia, altered bone microstructure, and an increased rate of bone resorption and overall fracture risk (11, 12, 20). Biochemical markers of bone resorption such as serum carboxy-terminal telopeptide of type-I collagen (CTX-1) and urine deoxypyridinoline (DPD) correlate with disease burden in patients with MGUS as compared with MM (21, 22), and MGUS patients have reduced levels of bone-specific alkaline phosphatase, a cell membrane-associated enzyme produced by osteoblasts, as compared to patients with non-malignant osteoporosis (23).

## Imaging techniques for multiple myeloma

Currently available imaging modalities allow characterization of lytic bone disease, bone marrow infiltration, bone mineral density (BMD), and extra-medullary disease involvement in MM. The primary purpose of skeletal imaging in MM has historically been identification of lytic bone disease, which allows classification of a patient as having smoldering or active disease, identification of bone lesions at risk of fracture and requiring acute management, and surveillance for new skeletal lesions based on patient symptoms and as evidence of disease progression. Recently however, the prognostic value of early identification of focal bone marrow involvement in MM, both at diagnosis and in response to antimyeloma therapy, has been evaluated.

### Identification of lytic bone lesions

Lytic bone disease is classically identified in MM using whole body radiography (skeletal survey, SS), which consists of conventional x-rays of the skull, spine, pelvis, chest, femora, and humeri, and was a component of the Durie-Salmon MM staging system (24, 25). SS remains the traditional gold standard for identification of lytic bone lesions, however standard radiography cannot detect early lytic bone lesions and therefore underestimates bone marrow involvement. Identification of lytic bone lesions using standard radiography requires loss of a minimum of 30% of trabecular bone volume (26), and a systematic review comparing imaging modalities for detection of lytic bone lesions concluded that low-dose, whole-body computed tomography, magnetic resonance imaging (MRI), and positron emission tomography (PET)-CT are all superior to SS for the detection of myeloma bone disease, except for in the ribs and skull (27). The updated International Myeloma Working Group (IMWG) criteria for the diagnosis of active multiple myeloma (8) reflect this data, and acknowledge that newer and highly sensitive imaging modalities, including low-dose whole-body CT and PET-CT may be used to satisfy CRAB criteria if lesions are >5 mm in size and even if lesions cannot be visualized by standard x-rays. At this time, the primary advantages of SS as a screening tool in MM are its low cost and widespread availability.

**Figure 1:**
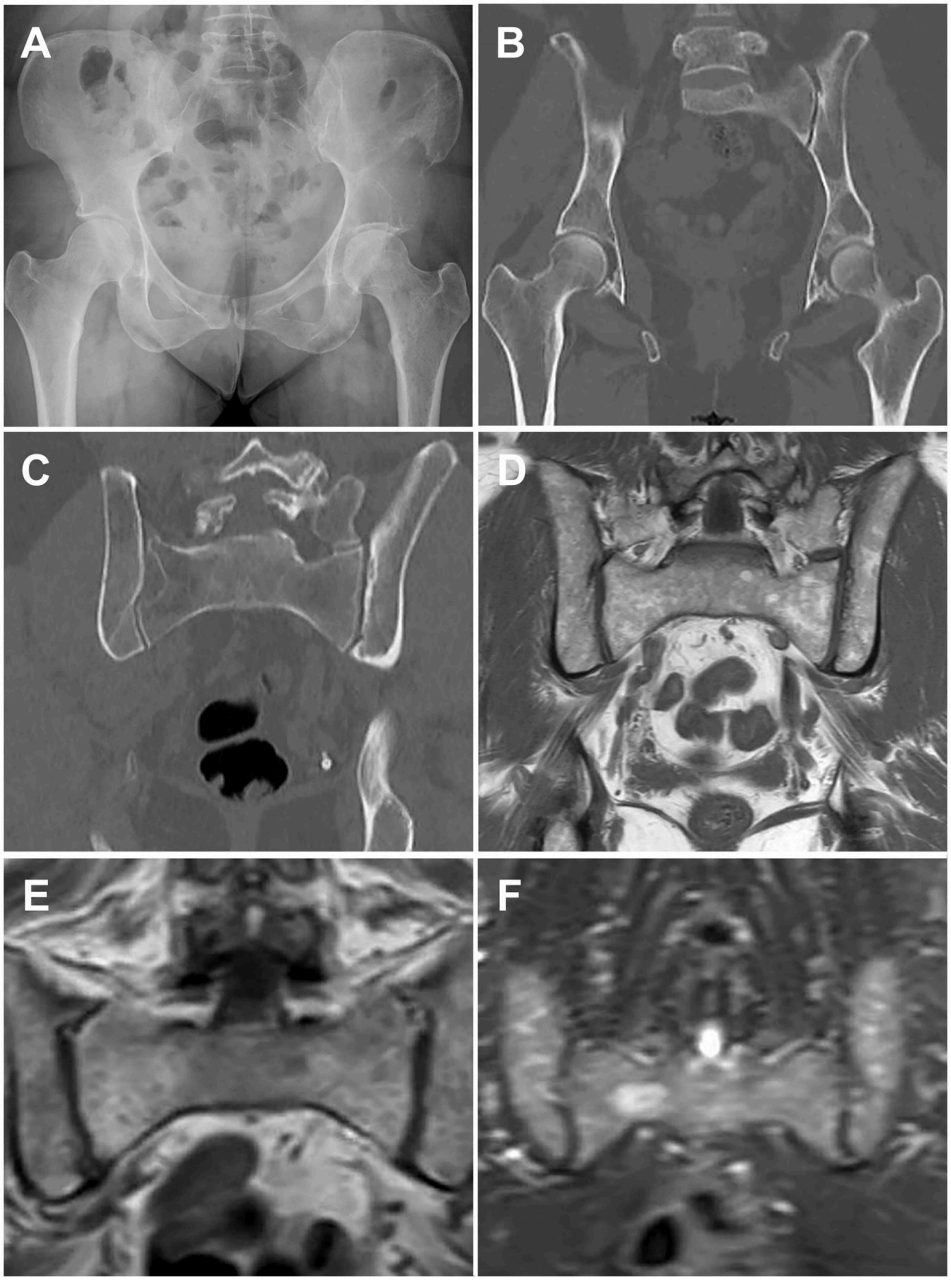
Paired images from patients illustrating different imaging modalities. Image pairs **(A,B)** and **(E,F)** are patients with active multiple myeloma. Image pair **(C,D)** is a patient with smoldering multiple myeloma. **(A)** Frontal pelvis radiograph demonstrates diffuse osteopenia with a dominant destructive osteolytic myelomatous deposit at the left supra-acetabular region as well as multiple smaller subtle lucent foci of disease. **(B)** Coronal reformat CT of the pelvis from a whole-body CT multiple myeloma protocol again demonstrates the dominant destructive left supra-acetabular lesion as well as multiple additional foci of smaller osteolytic myelomatous disease throughout the imaged osseous structures. Many of the smaller lesions identified on CT were occult on the comparison radiographs. **(C)** Coronal reformat CT of the pelvis from a whole-body CT multiple myeloma protocol demonstrates diffuse heterogeneity of the bone marrow including regions of mixed lucency and slightly increased density with a representative lucent focus at the superior aspect of the right iliac bone. **(D)** Coronal T1-weighted non-fat saturated image from a whole-body MRI multiple myeloma protocol demonstrates a diffusely heterogeneous appearance of the bone marrow without evidence for macroscopic myelomatous disease. **(E)** Coronal T1-weighted non-fat saturated image from a whole-body MRI multiple myeloma protocol demonstrates a diffuse micronodular pattern of myelomatous disease, also commonly referred to as a variegated or salt-and-pepper appearance. **(F)** Coronal STIR image from a whole-body MRI multiple myeloma protocol demonstrates diffuse heterogeneity of the bone marrow with a dominant hyperintense right hemisacral lesion compatible with macroscopic myelomatous disease.

Dedicated low-dose, whole-body computed tomography (WBCT) is an increasingly common imaging modality to screen for lytic bone disease in MM. Several studies have confirmed that WBCT is more sensitive than SS for the detection of lytic bone lesions, particularly in the axial skeleton, with some reporting that bone lesions were detected by WBCT in 20–25% of patients with negative SS, as well as fractures (28, 29). (Figures 1A,B provide examples of skeletal lesions identified on WBCT that are not visible on standard radiographs). In addition, WBCT can detect osteopenia and extraosseous disease. Based on these findings and the short scan time as compared to SS, many centers have moved to WBCT for initial screening for lytic disease. WBCT is less useful for monitoring response to therapy, as bone marrow lesions are poorly visualized with CT and lytic reactions persist after therapy. WBCT radiation doses vary according to individual institutions' WBCT protocols and are generally higher for WBCT than SS. However with the increasingly common adoption of low dose WBCT techniques the dose difference as compared to SS may be negligible. Additionally, the time required for radiologic review of WBCT images is greater than that required for SS, and clinically significant and insignificant incidental findings can be identified which may unnecessarily raise patient anxieties and lead to increased healthcare costs (30).

### Identification of focal bone marrow infiltration

MRI allows assessment of bone marrow involvement in MM and can reveal both diffuse bone marrow abnormalities and focal lesions. MRI has historically been coupled with SS to assess the spine and pelvis when determining if a patient has smoldering or active disease, for staging and response evaluation in patients with non-secretory MM, and in evaluation of suspected solitary plasmacytoma (24, 31). However, it has been reported that nearly 50% of patients with MGUS and MM have skeletal lesions detectable on MRI outside of the axial skeleton (32). In contrast to SS and CT, which are primarily used to identify cortical bone lesions in MM, MRI allows detailed evaluation of the bone marrow space and identification of varying patterns of bone marrow heterogeneity. Five patterns of marrow involvement in MM have been described and associated with tumor burden: normal marrow appearance, focal involvement (a focal lesion is defined by a diameter >5 mm), homogeneous diffuse infiltration, combined diffuse and focal infiltration, and a variegated pattern with inhomogenous marrow (Figures 1C,D compare CT and MRI imaging of bone marrow infiltration) (33, 34). High tumor burden is suspected in cases with diffuse hypointensity on T1-weighted images and diffuse hyperintensity on T2-weighted images, such as in Figures 1E,F. Cases with low tumor burden are usually associated with a normal MRI pattern (34).

Conventional MRI protocols are now increasingly used for whole body imaging (WBMRI), and revised IMWG diagnostic criteria for MM include the presence of more than one focal lesion (>5 mm) on MRI studies as a biomarker of malignancy (8, 34). MM lesions typically demonstrate low signal intensity on T1-weighted images, due to absence of intralesional fat and high signal intensity on fat-suppressed T2-weighted images, due to high cellularity and water content. Functional MRI techniques, such as dynamic contrast-enhanced MRI and diffusion weighted imaging provide further diagnostic sensitivity that can improve the detection of bone marrow infiltration. Dynamic contrast-enhanced MRI, which is not yet widely available in the clinic, assesses the distribution of contrast within and outside of blood vessels, providing data on vessel permeability that can be correlated with marrow angiogenesis, including the angiogenic response to therapy (35). Diffusion weighted imaging (DWI) measures the random motion of water molecules in tissue, providing information on tissue cellularity, cellular membrane integrity, and the extracellular space (36), and allows qualitative assessment of the bone marrow space in MM. Normal yellow marrow appears hypointense on DWI. As marrow cellularity increases, due to malignant infiltration or red bone marrow hypertrophy, signal hyperintensity increases corresponding to greater restricted diffusion (33).

The combination of WBCT with ^18^F-fluoro-deoxyglucose (FDG) positron emission tomography (PET-CT) provides an alternative method of visualizing bone marrow infiltration while also allowing visualization of total body tumor burden. Metabolic activity of lesions of interest is calculated based on FDG uptake in cells with high glucose demand and compared with standardized uptake values. CT images are then combined with PET images to provide anatomic localization. Importantly, hypermetabolic bone lesions can be identified in the absence of underlying lytic lesions. Active MM is FDG-PET-CT positive in the marrow space, although FDG-PET-CT is less sensitive than MRI for evaluation of diffuse marrow infiltration (36, 37). FDG-PET-CT is negative in patients with MGUS and SMM with low disease burden (38). Therapeutic response to treatment is characterized by a reduction or elimination of FDG accumulation in involved bone structures.

Multiple studies comparing whole body (WB)MRI or SS with MRI of the spine and pelvis to FDG-PET-CT in patients with active MM have demonstrated that MRI is superior to CT for detection of skeletal lesions (39). Results of studies comparing WBMRI to FDG-PET-CT, however, are mixed, and it is likely that the imaging modalities are of equal sensitivity (40), except for when evaluating the spine, where MRI is preferred (34). PET-MRI is a promising new hybrid technology, which in initial investigations appears to be at least as sensitive as PET-CT (41, 42).

An important caveat to these findings, however, is that standardized rubrics for interpreting MRI and PET-CT in MM continue to evolve (43, 44). In some cases the adoption of the imaging technology itself precedes the standardization of image interpretation, creating a challenging situation for treating clinicians. In addition, false positive bone marrow infiltrative findings are observed in both MRI and PET-CT studies. It is also difficult to distinguish red bone marrow from bone marrow infiltrated with MM on MRI with DWI, complicating interpretation of MRI bone marrow findings in younger patients (45), and PET-CT images can be falsely positive in the setting of trauma (including recent fracture), recent chemotherapy, radiotherapy and growth factors, and falsely negative following administration of high-dose steroids (46).

^18^F-sodium fluoride (NaF), a PET radiotracer which accumulates in both osteoblastic and osteolytic lesions, reflecting bone remodeling, has been investigated to a limited extent in MMBD (47). A recent small study prospectively compared SS, whole body MRI, FDG-PET-CT, and NaF-PET-CT in patients with newly diagnosed MM (48). MRI was superior to SS, FDG-PET-CT, and NaF-PET-CT. Detection of skeletal abnormalities by NaF-PET-CT was equivalent to SS, a finding consistent with other evaluations of NaF-PET-CT in MM patients (49).

### Imaging to assess disease response

Improvement in imaging technology has accelerated interest in the use of imaging to monitor disease response in MM. Historically, skeletal imaging with SS was performed at suspected disease relapse or in the setting of new skeletal symptoms with the goal of identification of progressive disease as evidenced by new lytic bone lesions. The utility of FDG-PET-CT for evaluation of disease response in MM has been extensively studied in both a prospective and retrospective fashion (46). Suppression of FDG-PET-CT focal lesions correlates well with disease response to therapy, and precedes resolution of lesions observed on MRI (50). Interestingly, it has been suggested that MRI-assessment of bone marrow infiltration, perhaps in combination with functional MRI techniques, may have utility as a measure of minimal residual disease (51).

### Imaging as a prognostic tool

The prognostic value of both MRI and PET-CT has been evaluated in MM patients. While the clinical significance of focal bone lesions that do not meet IMWG criteria for MM bone disease is not yet clear, serial WBMRI or FDG-PET-CT imaging can be used to follow the progression of these lesions. Focal bone lesions identified on axial MRI and not identified on SS correlated with overall survival in a large study of MM patients who received tandem autologous stem cell transplants. Sixty percent of patients studied had resolution of these lesions following treatment and superior survival (52). The presence of three or more FDG-avid focal lesions has been shown to be an independent predictor of overall survival (50, 53). Additionally, the persistence of three FDG-avid lesions after induction therapy for MM is associated with decreased overall survival (53). This data is supported by the IMAJEM study, a subgroup analysis of the Intergroupe Francophone du Myélome (IFM)/Dana Farber Cancer Institute (DFCI) 2009 trial (54). PET-CT and MRI were performed at diagnosis, following induction therapy and prior to maintenance therapy. Bone lesion identification at diagnosis did not differ significantly between imaging modalities. Normalization of PET-CT prior to initiation of maintenance therapy was associated with an improved 2-year progression free and overall survival. Interestingly, normalization of MRI before maintenance was not predictive of progression free or overall survival.

### Evaluation of bone mineral density

Age-related bone loss is traditionally characterized using dual-energy x-ray absorptiometry (DXA). While areal BMD is an established predictor of fracture risk (55), sequential measures of BMD are not routinely performed in MM and are challenging to interpret due to the heterogeneous BMD changes in MM (56). In addition, the revised IMWG criteria for the diagnosis of MM excludes osteoporosis in the absence of lytic lesions as sufficient to fulfill CRAB requirements of bone disease because many myeloma patients are elderly and have pre-existing osteoporosis and clarifies that bone densitometry studies are not sufficient to determine the presence of multiple myeloma (8). Despite this, it is important to recognize that the majority of systemic therapies for MM include glucocorticoids, which are themselves associated with increased fracture risk, and are included in the World Health Organization Fracture Risk Assessment Tool (FRAX) (55). Therefore DXA screening should be considered in patients undergoing active MM therapy who are not treated with bisphosphonates (due to intolerance or patient preference), or those requiring reinitiation of therapy who previously completed their bisphosphonate treatment.

Interestingly, MGUS is associated with skeletal fragility and MGUS patients have an increased risk of fracture, particularly axial fracture, as compared to age-matched controls (11, 18, 57). Recent studies employing quantitative computerized tomography (QCT) and high-resolution peripheral QCT (HRpQCT), imaging technology primarily used in osteoporosis research, have reported an overall increase in bone size with an increased endocortical area of the distal radius and diminished cortical thickness and bone strength in MGUS patients (12, 57, 58). The natural history of these findings in MGUS is not known, and it is not known if these abnormalities persist or change during progression to active myeloma.

## Selection of imaging modalities for MM

The International Myeloma Working Group (IMWG), European Myeloma Network, National Comprehensive Cancer Network (NCCN), the European Society for Medical Oncology (ESMO), and the British Society for Hematology Guidelines have all published guidelines to assist clinicians in choosing between available imaging modalities (24, 34, 46, 59–62). In all guidelines, WBCT is preferred for the diagnosis of lytic bone disease as compared to SS. The choice of imaging technology for patients without clear-cut myeloma bone disease, however, is more challenging, and dependent on available imaging technology and radiologic expertise. WBMRI, when available, provides excellent diagnostic and potentially prognostic information, however appropriate interpretation of the marrow changes that may be seen on images requires institutional experience. When WBMRI is not available, axial MRI should be performed when vertebral body involvement is suspected. PET/CT provides an excellent alternative to WBMRI when determining if a patient has active or smoldering MM. Key characteristics of the imaging techniques that are currently used most frequently for the identification of MMBD and clinical management of MM patients are summarized in the Table 1.

**Table 1:** Summary of advanced imaging modalities commonly used in the management of multiple myeloma.

	**Radiation dose**	**Examination time**	**Sensitivity for detection of focal bone lesions**	**Key references evaluating the efficacy of cross-sectional imaging modalities**	**Key references evaluating the prognostic utility of cross-sectional imaging modalities**
**Digital Skeletal Survey** (SS) (Chest; antero-posterior (AP) and laterval views of the spine, humera, femora; lateral views of the skull; AP view of the pelvis)	1.5–2.5 mSv	10 min. (Patients are repositioned during the examination)	Low, compared to cross-sectional imaging techniques.		
**Whole body low dose CT** (Vertex to mid- thighs), without iv contrast	4–7 mSv	5 min.	Superior to SS, particularly in the axial skeleton. Less sensitive	(28, 29)	
**FDG-PET-CT** (Vertex to mid-thighs)	Variable, based on institutional practice^*^	60–90 min. wait time following tracer injection, then 20 min. scan time	Similar to MRI	(36, 38–40)	(46, 50, 53, 54, 63)
**Axial MRI** (Spine and pelvis)	None	90 min.	Similar to PET-CT, limited by imaging field.	(32, 34)	(52)
**Whole body MRI** (Vertex to knees)	None	90 min.	Similar to PET-CT.	(34–36, 39, 40)	(51, 54, 64, 65)

* *Some institutional protocols obtain the CT portion of a PET-CT scan for the purpose of attenuation correction only*.

In conclusion, technology for assessing MM bone disease is rapidly evolving. Therapeutic clinical trials are beginning to routinely incorporate serial imaging assessments into their design, allowing investigators to evaluate the utility of these advanced imaging technologies to monitor response to treatment and disease progression.

## Author contributions

RS wrote the manuscript and reviewed the scientific literature. BH selected the images included in the figure, wrote the figure legends, and provided detailed comments and input.

### Conflict of interest statement

The authors declare that the research was conducted in the absence of any commercial or financial relationships that could be construed as a potential conflict of interest.
